# Brain Metabolism of Less-Educated Patients With Alzheimer Dementia Studied by Positron Emission Tomography: Erratum

**DOI:** 10.1097/01.md.0000484783.30769.90

**Published:** 2016-06-17

In the article “Brain Metabolism of Less-Educated Patients With Alzheimer Dementia Studied by Positron Emission Tomography”,^1^ which appeared in Volume 94, Issue 30 of *Medicine*, errors were detected in Table 1. The article has since been corrected online.
